# Modelling epidemiological and economics processes – the case of cervical cancer

**DOI:** 10.1186/s13561-024-00589-1

**Published:** 2025-02-22

**Authors:** Franziska Taeger, Lena Mende, Steffen Fleßa

**Affiliations:** Department of Healthcare Management, University of Greifswald, Friedrich-Loeffler-Strasse 70, 17487 Greifswald, Germany

**Keywords:** ABS, Agent-based Simulation, Bio-mathematical Models, Cervical Cancer, DES, Discrete Event Simulation, Markov, Modelling, System Dynamics

## Abstract

Different types of mathematical models can be used to forecast the development of diseases as well as associated costs and analyse the cost-effectiveness of interventions. The set of models available to assess these parameters, reach from simple independent equations to highly complex agent-based simulations. For many diseases, it is simple to distinguish between infectious diseases and chronic-degenerative diseases. For infectious diseases, dynamic models are most appropriate because they allow for feedback from the number of infected to the number of new infections, while for the latter Markov models are more appropriate since this feedback is not required. However, for some diseases, the aforementioned distinction is not as clear. Cervical cancer, for instance, is caused by a sexually transmitted virus, and therefore falls under the definition of an infectious disease. However, once infected, the condition can progress to a chronic disease. Consequently, cervical cancer could be considered an infectious or a chronic-degenerative disease, depending on the stage of infection. In this paper, we will analyse the applicability of different mathematical models for epidemiological and economic processes focusing on cervical cancer. For this purpose, we will present the basic structure of different models. We will then conduct a literature analysis of the mathematical models used to predict the spread of cervical cancer. Based on these findings we will draw conclusions about which models can be used for which purpose and which disease. We conclude that each type of model has its advantages and disadvantages, but the choice of model type often seems arbitrary. In the case of cervical cancer, homogenous Markov models seem appropriate if a cohort of newly infected is followed for a shorter period, for instance, to assess the impact of screening programs. For long-term consequences, such as the impact of a vaccination program, a feedback loop from former infections to the future likelihood of infections is required. This can be done using system dynamics or inhomogeneous Markov models. Discrete event or agent-based simulations can be used in the case of cervical cancer when small cohorts or specific characteristics of individuals are required. However, these models require more effort than Markov or System Dynamics models.

**Supplementary Information:**

The online version contains supplementary material available at 10.1186/s13561-024-00589-1.

## Introduction

Mathematical models are used in many sciences because they are faster, cheaper and less risky than reality [1]. Frequently, prognostic models are the only way to forecast future scenarios and make decisions early on, as waiting for the results would take too long and turn decision-making into a gamble [2]. However, not every model is suitable for a particular situation and the choice of model type is crucial for the validity of the results [3].

Many decisions in healthcare require the prediction of demographic (e. g. population, birth), epidemiological (e. g. incidence, prevalence, mortality) and economic (e. g. intervention cost, treatment cost, budget-impact, cost-effectiveness, quality-of-life) parameters in future periods. It is often insufficient to compare the costs and benefits of alternatives within one year, but the respective costs and utilities might incur over many different years. For instance, a vaccination program will require an investment in the year of the vaccination, but it will only yield results years later when the vaccinated person is immune to a disease. Thus, predicting the epidemiological and economic processes is crucial for deciding whether to start a vaccination program. Consequently, healthcare decision-making requires the selection of the most appropriate mathematical model for the epidemiological and economic process.

Cervical cancer (Cervix Uteri Carcinoma, CUC) is a complex disease requiring an extraordinarily thoughtful selection of the type of mathematical model. According to WHO, CUC is the fourth most common cancer in women globally, with around 660,000 new cases and around 350,000 deaths in 2022 [4]. About 88% of all cases [5] and about 94% of all deaths[4] due to CUC occur in low- and middle-income countries where screening and treatment are hardly available, particularly in rural areas. The modelling of this disease is more complex than for many other diseases. Firstly, caused by the human papillomavirus (HPV), i. e., it is an infectious disease and therefore the likelihood of being infected depends on the number of people who were previously infected and have become infectious, i. e., the infection cycle is dynamic and requires feedback loops between the previously infected and the current infections.

Secondly, the infection can (with a certain probability) lead to cancer, i. e., it is an infectious-disease that “behaves” like a chronic-degenerative disease. The WHO categorises CUC as a “malignant neoplasm” in the “noncommunicable diseases” category [6] although it is clearly a communicable disease. Modelling the processes related to CUC requires characteristics or features of chronic-degenerative and communicable diseases in the same model.

Thirdly, CUC is highly complex, as decades can lie between infection and death. On average, lesions will appear about 16 years after the infection, the transition from first lesions to cancer takes another eight years and untreated the patient can die within two years [7–10, 37], but in reality these values have a wide deviation [11]. Any intervention will have impacts that will become more pronounced over time.

Consequently, several studies using different mathematical models have attempted to model the epidemiological and economic processes of CUC in a population to support policy-making, for instance in implementing appropriate screening programs (methods, frequency, age groups), vaccination programs (age, frequency, efficacy) and treatment programs (radiation, surgery). In a recent study, Viscondi et al. [12], analysed Markov models for the economic evaluation of cervical cancer screening. Based on 38 models, they concluded that only “two studies justified the choice of model type […] and the overwhelming majority did not provide reasons or explain why the use of a Markov model was appropriate.” [12]. Without a rationale given in the paper, the type of model may be selected based on experience with a certain type of model rather than the specificity of the disease and research question.

It is not trivial to rationally select the most appropriate type of mathematical model, but it is crucial to obtain realistic results that are helpful to policymakers. Consequently, the objective of this paper is to support the selection of the most appropriate mathematical model to forecast epidemiological and economic processes. This paper will not consider models that address only incidence, prevalence and mortality, but only models that additionally focus on costs, budget-impact, cost-effectiveness and/or quality-of-life as policy-making takes place under conditions of scarce healthcare resources and require a thorough integration of economic processes in resource allocation decisions.

As previously mentioned, cervical cancer is a demanding case study particularly because it is an infectious disease that “behaves” like a chronic-degenerative disease. In the second section, we present an overview of mathematical models used in health economics in general. We then present the methodology of a literature review conducted to identify studies that apply mathematical models to cervical cancer. After presenting the results of this literature review, we discuss the advantages and disadvantages of each model type and conclude with recommendations for selecting the most appropriate model for epidemiological and economic processes of CUC and other diseases.

## Typology of Models

**Fig. 1:**
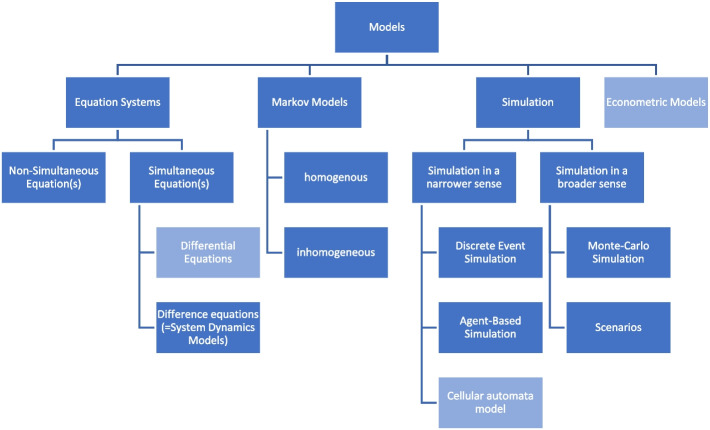
Typology of mathematical models in forecasting

Different models and typologies of models have been developed in order to forecast epidemiological and economic processes. Table 1 exhibits two different typology used in bio-mathematics [13, 14] and economics [1, 3]. For the analysis of this paper, we combine both approaches, but disregard models that are rarely used for the analysis of economic and epidemiological processes. Figure 1 shows the typology of models used in this paper.

**Table 1:** Mathematical Models for Epidemiological and Economic Processes. Source: [1, 3, 13, 14]

Bio-mathematics	Economics
• Statistical models o Regression o ML algorithms • Mathematical models o Deterministic models ▪ Ordinary Differential Equations ▪ Difference equations ▪ Partial Differential Equations o Stochastic models ▪ Stochastic differential equations ▪ Markov models • Simulation-based models o individual-based models ▪ cellular automata models ▪ agent-based models ▪ Discrete Event Simulation o population-based models	• Statistical models o Econometrics o Machine learning • Models of Operations Research o Optimizing models ▪ Linear Programming ▪ Game Theory ▪ Decision Trees ▪ Infinitesimal calculus o Prognostic Models ▪ Markov models ▪ System Dynamic Models ▪ Network techniques (e. g. CPM, MPM, PERT) o Simulation Models ▪ Simulation i. n. s ▪ Simulation i. b. s

To our knowledge, the first mathematical model of an epidemiological process was developed by En’ko in 1889. It focussed on the spread of measles in a school in St. Petersburg between 1875 and 1888, and it took 100 years until it was published in English [15]. Just a few years after developing this model, Ross published a mathematical model for malaria prevention based on data from Mauritius [16] which builds the foundation of the bio-mathematical models.

### Non-simultaneous Equation(s)

Many models consist of a set of equations that describe certain properties, such as the basic reproductive rate or the number of infections. The respective equations can be independent of each other (non-simultaneous equations) or form a finite set of equations for which common solutions are sought (simultaneous equations). The simplest form is a simple equation with a set of constants calculating an epidemiological parameter, such as the basic reproductive rate [17].

$${R}_{0}=\frac{m\cdot {a}^{2}\cdot {b}_{1}\cdot {b}_{2}\cdot {e}^{-\mu t}}{r\cdot \mu }$$, with.

**Table:** 

*R*_*0*_	Basic reproductive rate
*m*	Mosquitoes per human
*a*	Bites per mosquito per night
*b*_*1*_	Infection risk for humans
*b*_*2*_	Infection risk of mosquitoes
*r*	Recovery rate of humans
*μ*	Mortality of mosquitoes
*t*	Time

This first, simple model was followed by several other analytical models [18], but none of them were realistic enough to capture the complexity of infectious diseases with one equation or a set of independent equations. In reality, the parameters are not constants but interdependent and change dynamically. For instance, in the case of Ross’s malaria model, the number of mosquitoes per human (*m*) depends on the mortality of mosquitoes (μ), and the recovery rate of humans (*r*) depends (via semi-immunity) on the basic reproductive rate. In reality, therefore, we are not dealing with a simple equation, but with dynamic variables and a system of interdependent equations with time loops.

### System Dynamics Models

A system of dynamic simultaneous equations can be solved by ordinary or partial differential equations [13]. For most realistic problems, however, these models can only be calculated numerically by computing all parameters with discrete time steps. Consequently, it is also possible to express a system of simultaneous equations with difference equations. In the fields of economics and business administration, these models are called System Dynamics Models.

System Dynamics is a standard methodology of Operations Research with a wide range of applications in business administration and applied economics [19, 20]. System dynamic models are also frequently used in health economics whenever comparably realistic demographic, epidemiological and economic system parameters have to be analysed. The literature on System Dynamics applied to the healthcare sector is wide-ranging (e. g. [21–24]) and covers infectious diseases such as AIDS [25, 26] or Corona [27].

Forrester developed these models [28] for application in the industrial sector (e. g. storage and stock control), but the principle has been applied to many settings, such as environmental protection [29], urbanization [30], and diseases [19, 31]. System dynamics models express the dynamics of a system through a series of difference equations, which are usually solved numerically by calculating in fixed time steps (e. g. one day, month, or year). The following two equations present a highly simplified population dynamic. Population growth in period t (ΔP, births) depends not only on the fertility rate (f), but also on the population in this period t (P_t_). Consequently, the population in period t + 1 is the old population plus the new population, as a function of the old population. Parameters such as mortality, migration etc. are neglected here for the sake of simplicity, but the corresponding equations can easily be added.

$$\Delta {P}_{t}=f\cdot {P}_{t}$$

$${P}_{t+1}={P}_{t}+\Delta {P}_{t}$$, with.

**Table:** 

*P*_*t*_	Population in t
$\Delta {P}_{t}$	Births in t
*f*	Fertility rate

System dynamics models are calculated using modern IT and specialised application software [32]. They can become highly complex with thousands of compartments (states), interdependent states, and delay variables (e. g. for ageing) [33]. The precision of the computation depends on the time interval (Δt, e. g. years, months, days …). The model is particularly applied to infectious diseases [34] where the probability of infection depends on the prevalence rate, i. e., the proportion of the infected population within the total population. The most basic systems dynamics model (SIR: S: susceptible; I: infectious; R: removed) is even taught in business schools to future managers outside the healthcare system [19].

### Markov Model

The model most frequently used in health economics is the Markov model [35]. The Russian mathematician Andrey Andreyevich Markov (1856 – 1922) researched stochastic processes and developed the Markov chain as the most relevant model type for health care, as the health states (e. g. healthy, sick, dead) can be clearly distinguished and the transition probabilities do not depend on the decisions of policy-makers (e. g. mortality rate). For a Markov chain, it is assumed that the future states depend entirely on the current state, i. e., events that occurred before having no impact on the transition probability from state *i* to *j* (Markov property). The transition probabilities (*a*_*ij*_) form a transition matrix (A), and the vector of states at time *t* (*w*_*t*_) is calculated as

$${w}_{t}{\prime}={w}_{0}^{{\prime} }\cdot {A}^{t},$$

with $$A=\left(\begin{array}{cccc}{a}_{11}& {a}_{12}& \dots & {a}_{1n}\\ {a}_{21}& {a}_{22}& \dots & {a}_{2n}\\ \dots & \dots & \dots & \dots \\ {a}_{m1}& {a}_{m2}& \dots & {a}_{mn}\end{array}\right) \text{and }{w}_{t}=\left(\begin{array}{c}{w}_{1t}\\ {w}_{2t}\\ \dots \\ {w}_{mt}\end{array}\right)$$

**Table:** 

*a*_*ij*_	probability to change from status i to status j in Δt; i = 1,..,m; j = 1,..,n
*w*_*it*_	population in status i at time t; i = 1,..,m; t = 0..T
*t*	time
*T*	number of periods

The first applications of Markov models in health care appeared in the second half of the twentieth century, for example for the planning of clinical trials [36] or the allocation of health care resources [37]. They became very popular to forecast the development of chronic-degenerative diseases. In most cases, they assume a cohort which is followed during a number of periods, i. e., no additions are made to the population of the cohort. For instance, such a model can follow a cohort of individuals infected with the human immunodeficiency virus (HIV) over the next 20 years through various stages of infection (infected but antibody-negative; antibody-positive but asymptomatic; pre-AIDS symptoms, clinical AIDS; death due to AIDS) [38].

Markov chains can be homogenous or inhomogeneous. The vast majority of these models assume that the transition probabilities remain constant throughout the periods, i. e., they are homogenous, while other models re-calculate the transition probabilities (e. g. after each time interval). The new probabilities can be specified by the modeller (e. g. change of mortality rate assuming that a new medication is available at a certain time) or they are re-calculated based on the results of the last period. In the latter case, the Markov model becomes dynamic, as the probabilities change with time.

### Simulation in a narrower sense

Micro-simulation is a simulation in the narrower sense. While simulation in the broader sense means experimenting with existing models (e. g. different parameters and scenarios of existing Markov or System Dynamics Models), simulation in the narrower sense is a model built specifically for the simulation. It does not rely on other models but develops the (computer) model specifically for the scientific purpose. For epidemiological and economic problems, it is sufficient if the states change their values only at discrete points in time (Discrete-Event Simulation, DES), i. e. each event occurs at a particular point in time, but it is assumed no change occurs in the system between consecutive events. The computer develops an event list with all upcoming events (e. g. “patient will recover and become immune at t = 120”). The application of discrete event simulation modelling in healthcare is manifold, but the majority of models focus on service processes (e. g. queuing systems) in healthcare facilities, not diseases [39]. For realistic simulations, these events will be stochastic so micro-simulations are only useful for many runs [39, 40].

DES has the advantage that individuals are generated that can be related to each other. Unlike Markov or System Dynamics Models, we do not simply generate compartments with homogenous members (e. g. healthy population), but a set of rather independent entities within a compartment. This way, we can, for example, assign one individual newborn to one individual mother which is relevant for simulating the risk of mother-to-child transmission of HIV before and during birth as well as during breast-feeding [41]. However, traditional DES models assume that all entities of a certain type (e. g. mother, child) have similar characteristics.

DES offers an “intuitive and flexible approach to representing complex systems” [42] and is used frequently in health economics, in particular in health technology assessment (HTA). They can focus on one disease [43] or a bundle of diseases simultaneously [44], but more frequently it is used to model healthcare service processes [45].

One specific type of DES is agent-based simulations (ABS) where the agents (e. g. persons) interact with other agents and their environment. When an agent is created in a simulation, it is assigned a set of characteristics that will impact its behaviour. Furthermore, the likelihood of its decisions will depend on previous experiences (learning). In this way, it is feasible to distinguish individuals by their typical (e. g. risky sexual) behaviour without assuming that all individuals in a group are alike. ABS was used to simulate many different diseases and healthcare scenarios [46].

Simulations in a narrower sense can also be used to simulate the spatial diffusion of a disease, in particular by cellular automata models [47, 48]. However, the geographical dimension of disease spread has received less attention from health economists and will be neglected in this paper.

Simulation in a broader sense.

Markov and System Dynamics Models allow for the prediction of demographic, epidemiological, and economic parameters based on a set of constants. A major challenge is the uncertainty of these constants [49, 50]. Therefore, the modeller has to ensure that his results are reliable by re-calculating the models with different parameters. Experimenting with an existing analytical model can be referred to as macro-simulation or simulation in a broader sense. The constants can be deterministic (e. g. minimum, maximum, most frequent value) or stochastic. In the latter case, a random number following a specified distribution is selected for each stochastic constant, and the simulation is repeated multiple times to receive a reliable distribution of the result (e. g. cost). This form of stochastic simulation is called “Monte-Carlo” [51, 52].

As uncertainty is the norm in economics, all mathematical models in this science apply at least some instruments of simulation in a broader sense. Scenarios (“What happens if we alter a parameter?”) that result in a tornado diagram are the simplest form of simulation with an existing system dynamics or Markov model and are state-of-the-art for all health economic models according to the PRISMA criteria [53]. Stochastic experimenting with existing models has many advantages but is not commonly used. Thus, Monte-Carlo Simulations are a class of their own.

### Econometric or Biometric Models

Econometric or biometric models are frequently used to assess the correlation and causality between exogenous and endogenous variables. The main instrument is regression analysis, which requires extensive data sets. While these models have been used in general economics before, the first models predicting epidemiological and health economic parameters were developed in the second half of the twentieth century, initially mainly in healthcare planning [54, 55], later also for the demand and supply of healthcare services [56] and for specific diseases [57]. However, econometric models are more frequently used to analyse the impact of diseases on macroeconomic parameters [58] than to forecast the spread of the disease itself. The complex, non-linear, and sometimes non-monotonous relationship between interdependent parameters of the ecological and epidemiological systems makes applying econometric models rather cumbersome. Instead, they are often used to estimate parameters utilized by other models. Consequently, they are not considered in the analysis presented in this paper.

Most articles do not reflect on the rationale behind choosing a certain model. The main advice given by the literature refers to the distinction between communicable and non-communicable diseases. It is usually stated that homogenous Markov models should be applied for chronic-degenerative diseases, where a certain cohort is followed over a period of time and where probabilities (e. g. transition to another health state or mortality) remain unchanged throughout the process [35]. In contrast, communicable diseases require a model in which the “probability of a susceptible individual becoming infected at any one point in time (the force of infection) is related to the number of infectious individuals in the population” [59]. The models are nonlinear and “produce transmission dynamics that require specific consideration when modeling an intervention that has an impact on the transmission of a pathogen” [59]. Inhomogeneous Markov models, System Dynamics Models and micro-simulation models allow for this change in probability, but the literature does not sufficiently address the question of which model should be selected.

In addition, the terminology is not always clear. For instance, the International Society for Pharmacoeconomics and Outcomes Research (ISPOR) recommends the term “state-transition modelling” as an umbrella term for Markov model cohort simulations and micro-simulations as previously defined [35]. Others speak of “dynamic models” although Markov, system dynamic and DES-models are dynamic because they change over time.

Finally, there is no state-of-the-art model for a disease that is simultaneously infectious and chronic, such as CUC. Therefore, it is unclear which model should be used, which will be addressed in the following sections.

## Methods

We conducted a scoping literature review in order to determine the application of different models in forecasting epidemiological and economic processes related to CUC and the corresponding interventions based on the PRISMA statement [53]. The Database PubMed was used because it has the biggest overlap between epidemiology and economics. The results were confirmed by further analysis of Literature found in the databases EBSCO and Embase. In the first step, we selected publications on cervical cancer covering a wide range of interventions (e. g. screening strategies, vaccination strategies, curative services) where mathematical models were used. Furthermore, we focused on articles published between 2006 and 2023. This search period was chosen because of the introduction of the HPV vaccination in many low- and middle-income countries around 2006, which build as a starting point for many studies.

The following search strings were used:

- “Cervical cancer” or “Uterine cervical dysplasia” or “Uterine cervical neoplasm” or “Human papillomavirus” or “Papillomavirus infection” or “HPV” AND
- “Cost effectiveness” OR”Cost–benefit” OR “ICER” OR “incremental cost-effectiveness” OR “QALY” OR “quality-adjusted life years” OR “DALY” OR “disability-adjusted life years” AND
- “mathematical model” OR “dynamic policy model” OR “system dynamics” OR “Markov” OR “cohort model” OR “agent-based” OR “simulation”

The initial database search yielded 9,049 original research articles. Furthermore, we counted 12 research articles that cited 368 original papers in total, which were included in the review through cross-referencing (see Table 2). After the removal of duplicates, 9,074 sources remained. Of this total, 9,049 sources were original papers obtained through database screening, while of the 368 articles obtained through cross-referencing, only 25 review articles were found to be non-duplicates.

**Table 2:** Reviews of models for CUC. Source: own

Author	Year	Source	Number of studies reviewed	Models (acc. to paper)
Marra et al	2009	[68]	22	10 Markov, 1 hybrid, 11 dynamic
Jit et al	2011	[95]	6	1 Stochastic microsimulation, 1 State transition population model, 2 Markov models, 1 Transmission dynamic model, 1 Semi-Markov model
Fesenfeld et al	2013	[63]	25	16 static, 2 dynamic, 7 hybrid
Mendes et al	2015	[69]	153	149 static, 4 dynamic
Mezei et al	2017	[96]	19	11 microsimulation, 5 Markov, 2 semi-Markov, 1 decision tree
Viscondi et al	2018	[97]	38	All Markov; two studies justified the choice of model type
Mahumud et al	2020	[66]	12	9 dynamic, 2 static, 1 Markov
Malone et al	2020	[67]	15	5 Microsimulation, 3 decision tree, 3 Markov, 4 not reported
Linertová et al	2021	[65]	9	5 dynamic transmission, 3 Markov, 1 other
Shi et al	2021	[71]	14	4 cohort dynamic, 10 static (9 cohort and 1 individual)
Casas et al	2022	[98]	15	7 Markov decision model, 5 decision tree model, 2 microsimulation model, 1 semi-Markov simulation model
Wang, Sawleshwarkar et al	2024	[61]	40	11 primary system dynamics, 16 adapted system dynamics, 1 primary network-based model, 4 calibrations; 3 primary agent-based models, 5 calibrations

Articles that were not written in English, were not peer-reviewed (even if they fit the other criteria, e. g. [60]), and did not focus on the effectiveness or cost-effectiveness of different screening strategies and/or vaccination programs (e. g. purely medical articles) were excluded. Furthermore, articles that did not refer to a mathematical model were excluded and duplicates were removed. Finally, the findings of this study were compared to other reviews, even if the focus of these reviews differed from that of this study [12, 61–71]

For each article, information on the author, year, country or region of focus, rationale for selecting a model type, type of model, economic and/or epidemiological analysis, intervention, outcomes, study aim, and time horizon (comp. attachment) was collected. Most authors characterised their models based on the typology provided in Fig. 1, but in some cases, a re-assessment of the type of model based on the following assumptions was necessary.

- PRIME (Papillomavirus Rapid Interface for Modelling and Economics Model) is a Microsoft Excel based cohort static proportional outcome model. It is recorded as a non-simultaneous equations model.
- If a paper uses the (contradicting) term “microsimulation dynamic Markov model” without further explanation, it was assumed that the paper is a stochastic DES model.
- If a paper uses the term “individual-based microsimulation model” without further explanation, it was assumed to have utilised DES.
- If a type of model could not be attributed to a paper, it was labelled “unclear” (e. g. “hybrid with static companion” [72].

Furthermore, it was analysed whether the authors had given a rationale for selecting the type of model they chose. This question was answered with “yes” if the authors gave reasons for selecting a specific kind of model based on its appropriateness for answering the study question and its advantages in comparison to other types of models. Simply stating the purpose of the study without explaining why the respective model type is appropriate, was seen as insufficient. Similarly, simply referring to another paper or a model recommendation of an institution (e. g. WHO) was not recorded as a separate rationale unless the authors explained their model selection. It was also assumed that the statement that a model is deemed “user-friendly” was insufficient as rationale for selecting a certain type of model.

Finally, non-simultaneous equation models are static, i. e., they can be used to calculate costs and effects for different years, but the respective calculations for the individual years are independent. If a time horizon was given, the number was recorded even if its comparability to the time horizons of other models was limited.

## Results

We retrieved 71 original articles (Attachment: Key characteristics of the models) of which the vast majority of articles (66) were published in journals of Public Health or Medicine. Two papers were published in methodological journals (Health care management science [73], EURO Journal on Decision Processes [74]), and three in journals related to health economics and planning (International journal of technology assessment in health care [75], Health policy and planning [76], Value in health [77]). In comparison to the works published in medical and public health journals, the methodological and economic papers were older, i. e., the most recent methodological paper was published in 2017, while the one most recently published paper in medical or public health journal was published in 2023. Furthermore, 12 systematic reviews were screened (see Table 2). Of these 12 review papers, only one was published in a health economic journal (Pharmacoeconomics, [68]), while all others were published in journals on Medicine and/or Public Health.

In total, 20 papers were conducted in low and lower-middle-income countries, 45 were situated from upper-middle or high-income countries, based on the classification of the World Bank [78], while the remaining papers had a more global perspective. The USA was found to be the focus country of five articles and thus was the most represented country in this review. However, a focus on CUC in Uganda, China and India could be recorded in four papers for each country, although some of the papers, particularly ones focused on Uganda, were found to be rather similar.

The majority of papers used System Dynamics Models (23), followed by homogenous Markov models (15) and DES (12). Nine papers applied non-simultaneous equation models with several independent equations, usually compiled in a spreadsheet. Seven papers used an agent-based simulation approach, of which only one focused on a setting outside of a high-income country. Several papers used a Monte Carlo simulation for scenarios and sensitivity analysis, while the original models underlying their research were system dynamics or Markov models. Four papers combined system dynamics with another type of model (i. e. agent-based: 3; Markov: 1), thus creating a hybrid model (multi-method modelling [79]). No application of inhomogeneous Markov models was found in this review. The more sophisticated models (i. e. agent-based, hybrid models) were focused on upper-middle or high-income countries, with the exception of DES, which were tailored for Uganda [70, 76, 80, 81] and Nigeria [82].

Most models supported the analysis of vaccination strategies (31) or compared vaccination to screening programs (21). Screening programs alone (e. g. self- vs. institutionalized sampling) were addressed by 14 papers. Five articles focused on other properties, such as the role of acquired immunity or treatment. Cost-effectiveness in different forms (e. g. cost per QALY, cost per year of life saved, cost per life year lost) was found to be the focus of 58 papers, while 10 papers concentrated only on effectiveness, irrespective of the associated costs. Thus, these papers primarily addressed the epidemiological dimension without including an economic perspective. Three papers followed a different research focus, such as the trade-offs between equity and efficiency.

An assessment of the quality of the models was found to be challenging. Only one paper [74] referred directly to health economic standards, such as the Consolidated Health Economic Evaluation Reporting Standards, CHEERS [83]. Most notably, the rationale of the model (CHEERS checklist item 16) was grossly neglected by 56 (79%) of the papers, even though the CHEERS checklist calls for a detailed description of the model and an explanation of why this type of model was used. The rationale behind the choice of model is usually detailed in the original research papers, while other papers build on them by making minor structural changes or merely changing parameters. Wang et al. categorise the original models as “primary”, the models with minor adjustments as “adapted”, and the models adopting a simple change of parameters as “calibration” models [61]. In the case of adapted structures, most articles refer only to the original source without further explanation of the adapted model. In the case of calibration (e. g. utilising the same model for another country), no paper was found to explain the model sufficiently.

The selection of the type of model differs a lot, while most models do not give a reason for their decision. This is particularly true for papers using public domain models, such as PRIME (Papillomavirus Rapid Interface for Modelling and Economics) [84–89] or CERVIVAC [90]. This review found that most publications referring to these readily available models, did not include a description of the model nor of the rationale for the model selection.

However, some papers included detailed explanations as to why they selected a certain type of model. For instance, Olsen and Jepsen write extensively why they used an agent-based model: “Agent-based models are well suited for modeling sexually transmitted diseases because each individual of the population is directly represented in the model, thus allowing the model to capture individual heterogeneity in sexual behaviour and the herd-immunity effect of vaccinations” [75]. Similarly, explanations were given for the four agent-based models [75, 91–93]. The majority of non-simultaneous equation models, system dynamics and Markov models neglected this explanation.

Other relevant CHEERS checklist items include the perspective (8), time horizon (9), discount rate (10), selection and valuation of outcomes (12, 13), the measurement and valuation of resources and costs (14) as well as the handling of uncertainty (20, 24). The perspective, such as health insurance, patient or society, was grossly neglected by most papers; discount rates were usually given. Fifty-seven of the articles stated the time horizon. Markov models are cohort models, so “lifetime” was the most frequent time span given(19). Other models analysed scenarios for up to 100 years. For Markov, system dynamic and micro-simulation models, a time horizon should be given, but some papers (one on agent-based models, one on DES, seven on system dynamics, two on Markov models) ignore this criterion of good health economic research. Most papers that provided a time horizon analysed vaccination programs, while papers focussing on screening alone were frequently found to provide no or only a “lifetime” horizon.

The majority of papers reviewed used one or more instruments of scenario techniques or sensitivity analysis, e. g. macro simulation incl. Monte Carlo simulations. However, the choice of adopted methodology was rarely explained, i. e., no rationale was given as to why a stochastic technique was applied to a larger cohort. One exemption is the paper by Flessa et al., specifically addressing uncertainty, in which CUC was used as a case study to exemplify types of uncertainty and instruments to model it [74].

## Discussion

### Sample

In this paper, we present an analysis of different mathematical models forecasting the epidemiology and economics of CUC. As shown in Table 2, the 71 papers included in this analysis constitute one of the broadest samples. Other reviews have a different focus and/or use a different terminology. For instance, Mendes et al. distinguish static and dynamic models, the terms “Markov”, “system dynamics”, “agent-based” or “simulation” however, are not mentioned in the review paper on “Systematic review of model-based cervical screening evaluations” [69].

Other review papers exclude certain models from their analysis. For example, Wang et al. neglect non-simultaneous equation models [61], i. e., they ignore several papers based on public domain software for calculating the cost-effectiveness of interventions targeting CUC. For instance, the “Papillomavirus Rapid Interface for Modelling and Economics” (PRIME) is a non-simultaneous equation model to estimate the impact of cohort vaccination. It is static and “herd effects and cross-protection” are ignored [84]. The respective equations are shown in Appendix 1 of the paper of Jit et al. [94]. The model is widely used [66, 84, 85, 87–89] although it does not allow for inclusion of herd immunity effects. Wang et al. do not state why this branch of papers was neglected, but one reasons could be that they challenge their relevance for scientific analyses.

### Terminology

One common challenge among all meta-analyses and this review is that there is no universally accepted and applied terminology. Above all, sometimes no distinction is made between the terms micro-simulation, DES and agent-based simulation. For instance, Van der Ploeg et al. define the term(s) as: “A micro simulation model is a special type of a discrete-event system in which life histories of hypothetical individuals are simulated over time by means of a computer program. Each individual is represented in the model by a number of characteristics, some of which remain constant (for example, sex and date of birth), while others change during the simulation (for example, infection and disease status, and number of sexual partners). Changes in characteristics are the result of discrete events that occur during the life of the simulated individuals, like starting and ending sexual relationships, acquiring infections, and cure. Most of the events are stochastic: their occurrence in the life of an individual is determined via probability distributions based on data from the total population.” [99]. Particularly the second part of the definition is more appropriate for an agent-based simulation where the history of events and the corresponding learning, that leads to changes in the individual’s characteristics, are frequently seen as the main feature of this type of model [100]. Another paper refers to”individual stochastic system dynamics”, but defines the simulation as a stochastic DES [61]. This incoherent understanding of the terminology is further reinforced by other papers referring to these definitions without reflection, such as the one by Matthijsse et al. [92].

**Fig. 2:**
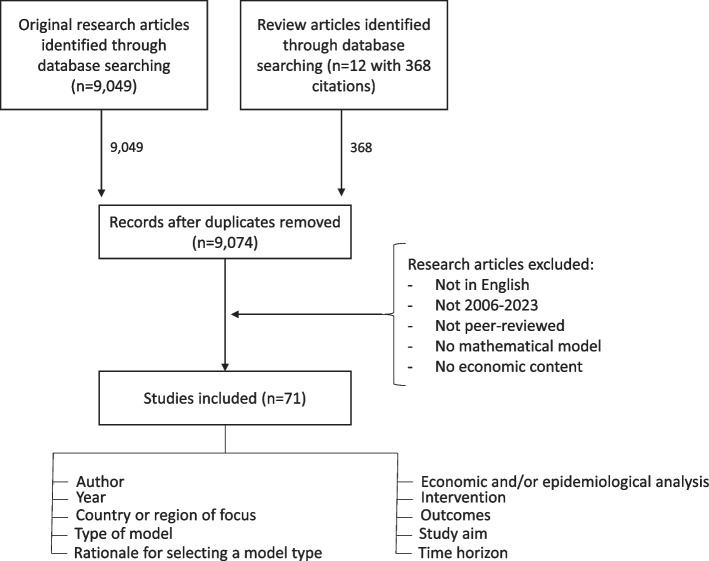
Study Flow Chart. Source: own

Some papers use the term “network models “ when addressing a model that is referring to a sexually transmitted disease like CUC, as it is a kind of disease that is transmitted within a network of sexual relationships, e. g.[101]. Based on the typology given above (see Fig. 1), these models are DES or agent-based simulations, in which the network or the likelihood of having sexual intercourse with a person within the same network is defined as a characteristic of the respective individual. Consequently, it is questionable whether network models are a class of their own within the typology of models Fig. 2.

### Modelling Cervical Cancer

**Fig. 3:**
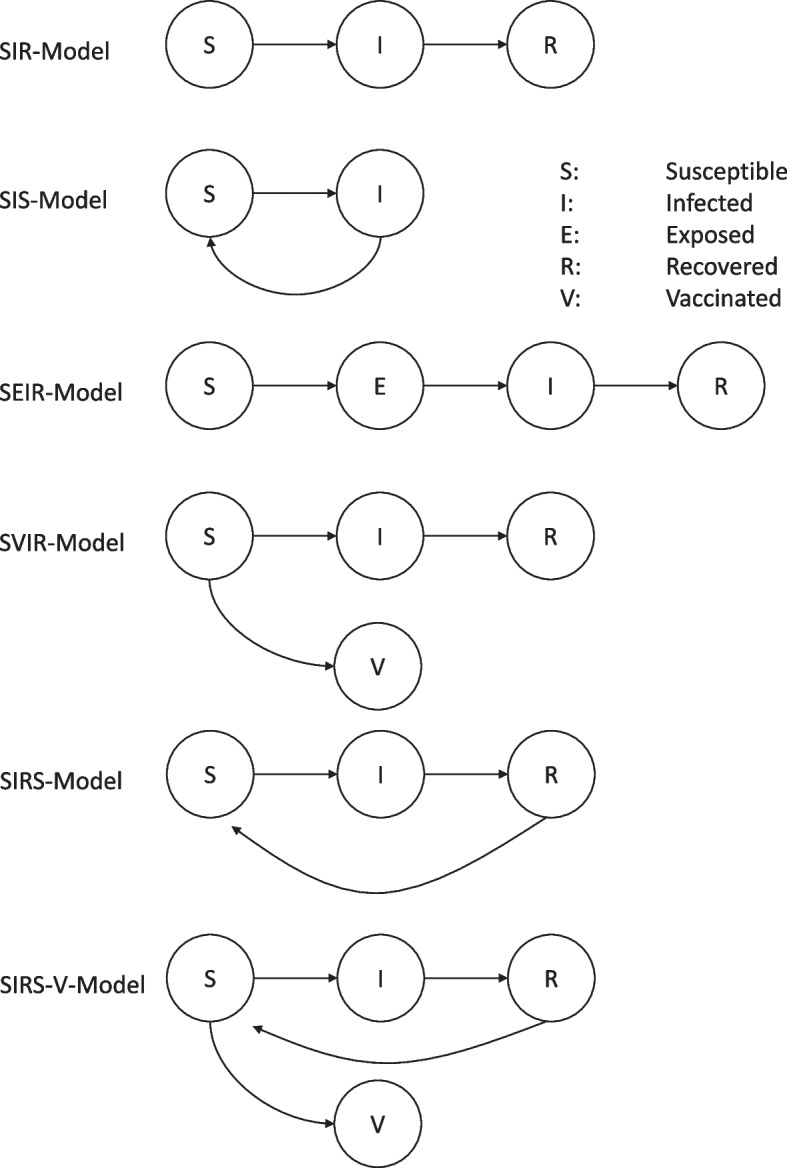
Models for infectious diseases. Source: [19, 61, 102]

The terminology of models is particularly complex in the case of cervical cancer, as the disease is infectious and can be chronic at the same time. Dynamic models of infectious diseases can usually be represented by one type, as shown in Fig. 3. The simplest model version has three health states, i. e., susceptible (S), infected (I), and recovered (R) [61, 102], which are established as separate compartments in the model (Fig. 3). In some cases, the agent (e. g. virus, bacteria) is cleared after some time and the patient returns to status S. SIS- and SIR-models can be combined, i. e. some of the infected individuals clear and become susceptible to infection again, whereas another segment of the infected population will recover and remain immune. The SIR-model can be generalized to a SEIR-model in which the special compartment “exposed”, consisting of people having been exposed to the agent but who are not yet infected is introduced. The SVIR-model, finally, introduces the compartment “vaccinated”, for the vaccinated population that cannot be infected. In some cases, immunity does not last life-long, so the recovered person will return to the compartment “susceptible” (SIRS). Finally, some models combine SIRS with SVIR and allow two types of immunity. A short-term form of immunity attributed to recovered infection and a long-term immunity due to vaccination.

**Fig. 4:**
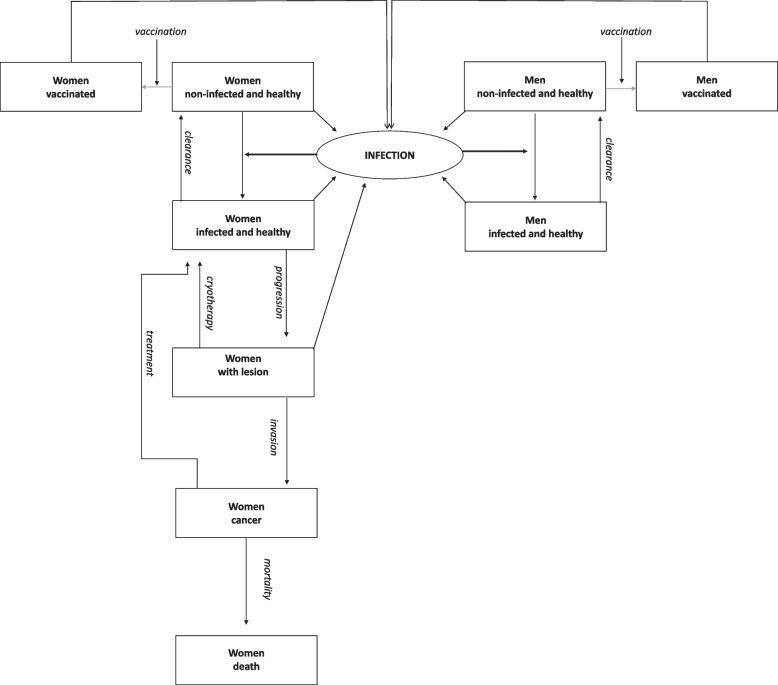
Cervical Cancer Model. Source: own, basa on [113]

A realistic model of CUC (comp. Figure 4) is more complex and combines the models given above. The model-builder has to decide:

- Women or men: The virus is usually transferred from one sexual partner to the other through intercourse, but only women develop CUC. Some models do not consider the epidemiology of the male population (e. g. [103]).
- Stems: Several stems of the papillomavirus exist simultaneously. Most models exclude the variety of stems and model a “pattern stem”, while other models consider different stems at the same time (e. g. [104]).
- Infection: Some models start with a cohort of infected and follow them for the rest of their lives (cohort models, e. g. [103, 105]), while other models explicitly model the infection, e. g. in the studies of Flessa et al. and Olsen et al. [74, 75]. The risk of infection depends on the prevalence of HPV in a population, i. e., on the incidence of former periods. Thus, only dynamic models can consider the infection profoundly. Homogenous Markov models have to assume that the infection rate is constant [105]. For very short forecasting periods, assuming a constant infection rate is acceptable, for longer periods it is misleading.
- Age: CUC is a sexually transmitted disease, and sexual activity is found to be dependent on the age of the sexual partners. Some models distinguish between age sets, while cohort models usually do not (e. g. [77, 106]).
- Sexual behaviour: Agent-based models can distinguish between individuals and their sexual behaviour, i. e., long-term partnerships, short-term relations, and coitus frequency that have an impact on the infection probability. Including this distinction is feasible but more complex for System Dynamics Models (e. g. [74]), and is not possible in other model types.
- Vaccination: If the model analyses the impact of a vaccination program, separate compartments of vaccinated and immune populations are established. The efficacy of the vaccine can be modelled by transferring only a certain percentage of the vaccinated to the compartment of the vaccinated (e. g. [75, 107]).
- Clearance: The immune system can defeat the virus, i. e., a share of the population of the compartment of the infected and the healthy will be transferred back to the compartment of the non-infected population. Some models include this process of clearance (e. g. [77, 92]), however, many do account for it.
- Screening: Different models analyse different screening strategies, such as self-testing devices, point-of-care tests (e. g. visual inspection with acetic acid [108–110]), or laboratory tests (e. g. pap smear) [111].
- Treatment: Pre-cancerous lesions can be treated by cryotherapy [112, 129], but cancer treatment requires surgery and radiation. After a lesion is removed or a patient is successfully treated, the patient can be transferred back to the infected and healthy population compartment. The efficacy of interventions can be also modelled.
- Transition: On average, an infected woman will develop a pre-cancerous lesion 16 years after infection, and it takes another 8 years on average for the lesion to develop into cancer [7–10, 37]. All models must recognize this long-term transition process as a chronic disease.
- Stages of cancer: Some models distinguish between different stages of cancer as defined by the Fédération Internationale de Gynécologie et d'Obstétrique (FIGO). The stage determines the mortality rate [9, 75]), however, several models do not take these differences into account and work with average values.

To summarize, this review found that CUC is a highly complex disease requiring a complex model because of the two aspects of the disease: infection and chronicity. Simple models cannot be used to simulate a complex epidemiological process. Consequently, simple models must not be used for complex economic processes related to disease spread and progression. However, cervical cancer is not the only disease with this dual characteristic. A number of viruses are known to cause cancer, such as Epstein-Barr virus (lymphoma), Hepatitis B virus (liver cancer), Hepatitis C virus (liver cancer), Human immunodeficiency virus (e. g. Kaposi sarcoma, lymphoma), Human herpes virus 8 (Kaposi sarcoma), Human T-lymphotropic virus (leukaemia/lymphoma) [114]. These dual diseases pose a challenge even for the compilation and evaluation of statistics, as the World Health Organization classifies all of them as category II diseases (non-communicable diseases), although their origin is infectious and should be considered category I diseases (communicable, maternal, neonatal, and nutritional diseases). Other examples include the links between pathogenic fungal infections [115] or aflatoxins (*Aspergillus flavus* and *Aspergillus parasiticus causing liver cancer*) [116] and cancer development and pathogenesis [117]. In all of these cases, it is difficult to model the pathway from the contact with the agent (virus, fungus, …) to cancer, as it is a multi-cause-multi-effect model of epidemiology [118]: Contact with the agent can be completely harmless or cause a variety of diseases. At the same time, however, the disease can be triggered by other causes. Currently, there is no gold-standard for the best model simulating such complex diseases, but this review could identify certain indicators to assess which type of model is most appropriate for infectious chronic disease.

### Selecting a model

Table 3 summarizes the pros and cons of the different models reviewed in this paper, as well as their applicability to different epidemiological situations. Non-simultaneous equation models are the simplest models which can be computed with a calculator or a spreadsheet. With this structure, the models are simple but can be misleading. For instance, an intervention (e. g. vaccination) will not only protect the vaccinated girls but also reduce the likelihood of boys being infected in the future. Thus, vaccinating a girl today increases herd immunity and consequently reduces the infection risk for all other individuals (including boys and girls, men and women). Thus, the efficacy of one vaccination goes far beyond the direct impact on the vaccinated girl, but this cannot be reflected in static models. What Jit et al. write about the PRIME model applies to all non-simultaneous equation models: “We did not consider indirect effects (herd protection), thus the model provides a conservative lower bound on vaccine effect “[94].

**Table 3:** Applicability of epidemiological models. Source: own

	**Non-simultaneous equation**	**Markov**	**System dynamics**	**Monte-Carlo**	**DES**	**ABS**
Workload	Low	Medium	Medium	Medium	high	Very high
Computation	Calculator, spreadsheet	specific software	Specific software	Partly included in specific software	simulators	Simulators
Feedback-loops	No	No	Yes	Depends on model	Yes	Yes
Data requirement	Low	Some	Some	Some	Some	Comprehensive
Forecasting cohort	No	Yes	Yes	Yes	Yes	Yes
Small numbers	Yes	No	No	No	Yes	Yes
Individuality	No	No	No	No	partly	Yes
Main use	“Quick and dirty” assessment	Forecasting chronic-degenerative diseases and cohorts	Infectious diseases	Sensitivity analysis for Markov and system dynamic models	Smaller numbers, e. g. begin of epidemic	Smaller numbers with individual behaviour

Markov models require the definition of states and transition probabilities, but the calculation itself is usually based on standard software (e. g. TreeAge). System dynamics models usually require the definition of difference equations, which can be much more challenging. Standard software (e. g. VenSim) is available, but many researchers prefer to program the model themselves in a high-level general language, such as D + + , Delphi, Java etc. All of these models can be combined within a Monte-Carlo simulation, but this makes the work even more challenging. The amount of work required to develop DES’s is quite high, although standard simulation software for DES is available [39]. The highest workload is required for agent-based simulations, even if a software is available [79].

Non-simultaneous equation and homogenous Markov models do not allow a feedback loop, i. e., the results of period t have no impact on transition probabilities in period t + i with i ≥ 1. Inhomogeneous Markov chains allow the recalculation of the transition probabilities every period, coming very close to the simulation possibilities of System Dynamics Models. Merely the calculation methodology (matrix multiplication vs. difference equations) differs. The other models allow for the introduction of feedback loops, e. g. the infection probability is a function of former infections. Agent-based simulations even allow for adjusted behaviour through “learning”, i. e., individuals can change their behaviour based on past experiences.

The need for empirical data is rather low for non-simultaneous equation models. Markov-models require data on the population of each compartment as well as the transition probabilities. As most cohort models simply follow the transition of a cohort through the stages (e. g. infected → lesion → cancer → death), the data requirement is not very high, e. g., the average duration in a stage is sufficient to calculate the transition process. System dynamic models can be very extensive and require a lot of data, e. g. on infection and (sexual) behaviour. However, these models can also be rather simple without large data requirements. ABS and DES can become quite strong with a large data requirement, while smaller models are also feasible. Data availability is a challenge, particularly in low-income countries with less developed health information systems. What Jit et al. wrote about HPV vaccinations applies to many models: The “assessments of cost-effectiveness of HPV vaccination often use complex models with data and expertise requirements that are prohibitive in many settings. Application of such models to resource-poor settings might require dependence on external consultants, which can sometimes restrict the involvement of local analysts and policy makers and, consequently, the effect that these results have on local decisions.” [94]

The purpose of the models differs as well. Most models allow the prediction of future events, while non-simultaneous equation models are static. They allow the calculation of cases, costs or reproductive rates, but the results represent a kind of equilibrium, not a process. However, all other models are suitable for simulating future events. A key difference is the capability of the model to simulate smaller numbers. Non-simultaneous equation, Markov and System Dynamics Models analyse cohorts or compartments, but do not distinguish between individuals. Consequently, Monte Carlo simulations building on these models do not consider individuality. This type of prediction is appropriate for the “average person” in a cohort or compartment without considering individual behaviour or probabilities. At the beginning of an epidemic, the spread of the disease is highly stochastic, as each infection is a stochastic process. While cohort models use averages, DES and ABS consider each infection as a separate event and the respective variable as binary, i. e., infection or no infection. Even with a basic reproductive rate greater than one, an epidemic could end soon after the first case occurs, simply because the infection is a stochastic process and this patient might not infect another person. Thus, the beginning of an epidemic and a small cohort of infected individuals will require stochastic models, i. e. DES or ABS. Monte Carlo simulations based on other models cannot account for individual infection risks and therefore also focus on larger cohorts in the later stages of an epidemic.

To summarize, this paper found that each type of model has its advantages and can be used under certain conditions and with certain research objectives. Non-simultaneous equation models are easy-to-use and rapid instruments that allow a simple epidemiological and/or economic assessment of the spread of a disease. For example, the basic reproduction rate can be calculated as an average for a certain population. Markov models can predict the epidemiological and economic processes of a cohort of infected individuals through the different stages of the disease and in particular allow for the impact of screening programs to be analysed. For short predictions (e. g. 2 years), newly infected individuals have only a limited impact on the risks of infection of other people, so the risk of infection can be assumed to be constant. In this case, Markov models can also be used to predict the entire process shown in Fig. 4. For longer time periods, the risk of infection changes because earlier infections will increase the risk of later periods. Therefore, an inhomogeneous Markov chain where the respective probabilities are recalculated every period would be required.

In their “systematic review of Markov models for economic evaluation of cervical cancer screening”, Viscondi et al. conclude that “a Markov model may be suitable if the objective of the study is to assess alternative screening strategies in a setting in which disease prevalence is constant”, but they see the “limitation of the closed population model (such as a Markov cohort model) […] that it may predict an increased cancer incidence compared with an open model”. If the analysis incorporates the effect of an HPV immunization program, the ideal model would be a dynamic model that follows an entire population, allowing for the evaluation of the impact of herd immunity (i. e., indirect protection of susceptible individuals by a significant proportion of immune individuals in the population) [12].

System dynamics models are very flexible and allow a high degree of interdependent equations with feedback loops. Infection can reflect a wide range of variables (e. g. infectivity, coitus frequency, desire for partnership, length of partnership, multiple sexual partners, vaccination status), making the model quite realistic. However, these parameters are not always available, so the models show details without reliable data. For example, the coitus frequency is hardly available in statistics of many countries and the desire for partnerships can only be estimated. System dynamics models can predict long periods. This is particularly important for a disease like cervical cancer where (on average) 24 years lie between infection and cancer [7–10, 37], and 30 or more years can lie between vaccination and avoided cancer.

A number of system dynamics and Markov models use macro simulations to account for different forms of uncertainty. The first form of uncertainty is the uncertainty of parameters, such as interest, time preference rate, infectivity, partnership pattern etc. In a deterministic macro simulation, the model is re-calculated once for each deterministic parameter, while in the Monte Carlo simulation, the model is re-calculated many times with random numbers of a given distribution. The second form of uncertainty is structural uncertainty, i. e., the model-builder is not sure whether his equations are correct and simulates several possibilities of the equations and analyses whether the results change significantly. Often this becomes “modelling for insights, not for numbers” [119], i. e., we analyse whether structural changes have an impact on policy-decisions.

DES and ABS create individuals and each event (e. g. infection, vaccination, screening, cryotherapy, treatment, death) is generated for exactly this individual. This is very helpful if the number of individuals is small (beginning of infection) or if the individuals have specific characteristics (ABS). Theoretically, everything that can be done with Markov or System Dynamics Models can be also done with DES and ABS, but it requires much more effort and computing time, particularly for longer time series and larger groups of individuals. The advantages of DES and ABS lie in their flexibility and precision. At the same time, they offer probability distributions as results instead of point estimates. What Simpson writes about the comparison between Markov and DES models, could also be applied to system dynamics and ABS: “The DES model predicts the course of a disease naturally, with few restrictions. This may give the model superior face validity with decision makers. Furthermore, this model automatically provides a probabilistic sensitivity analysis, which is cumbersome to perform with a Markov model. DES models allow inclusion of more variables without aggregation, which may improve model precision. The capacity of DES for additional data capture helps explain why this model consistently predicts better survival and thus greater savings than the Markov model. The DES model is better than the Markov model in isolating long-term implications of small but important differences in crucial input data” [120].

This paper has demonstrated that a large number of papers have been published using very different types of mathematical models to analyse epidemiological and economic processes of cervical cancer. The variety of models is appropriate, as different researchers ask different research questions, have different assumptions and purposes of the models, and analyse different interventions (e. g. screening or vaccination). Thus, it is difficult to compare the models, i. e., “existing analyses have been done with various model types, ranging from simple static models that only consider direct effects, to complex individual-based transmission dynamic models. This variation in model types restricts the comparability of their results because different model types rely on different simplifying assumptions” [94]. As previously explained, the majority of papers do not provide a rationale for a type of model, nor do they follow the standards of health economic evaluations [35]. Without scientific proof, the authors developed the “feeling” that some model-builders have used the type of model with which they are most familiar, instead of the type of model best suited to the specific purpose. As Abraham Maslow noted: “For the man with the hammer, everything looks like a nail”[121, 122], we might state, “For the scientist with Markov models, everything looks like a Markov chain”.

There are a number of limitations of this paper. Firstly, only papers published in English were considered, and it is likely that some papers and/or models are published in other languages, which were not assessed. Secondly, some papers did not include sufficient information about the models. In some cases, it was only possible to “guess” what the methodology was like and/or what changes were made based on another model. Although this was done by two experts, it is possible that some of the superficial modelling information was misinterpreted. Thirdly, the terminology used by papers is not always consistent. For instance, the term “dynamic” is used by some authors to express a System Dynamics Model, while DES or ABS are certainly dynamic as well. It was attempted to accommodate for this imprecision in terminology by reviewing a broad sample of papers of research worldwide from the past 20 years, providing a comprehensive systematization of the state of the art in modelling epidemiological and economic processes in cervical cancer.

## Conclusion

Based on these findings, different types of models are appropriate for cervical cancer, but their applicability depends on the time horizon, the purpose of study, the stage of the epidemic and the efforts that can be invested in the model. Homogenous Markov models are rather simple and appropriate to model a cohort of newly infected for a lifetime. This allows the modelling of the impact of screening programs on epidemiological and economic outcomes under the assumption that screening will not have a (major) impact on the likelihood of infection. Instead, vaccination programs will reduce the number of new infections. Thus, they require a feedback loop, transferring individuals from cases (= former infections) to new infections. This can be done by system dynamics, discrete event simulation and agent-based simulations. System dynamics is the simplest dynamic model allowing for such feedback. It can be used for bigger cohorts and long-term forecasts where the role of individual probabilities is reduced by the huge numbers in each compartment.

The advantage of a stochastic DES is that the likelihood of an infection of an individual can be modelled. This is particularly important if a new agent or stem is introduced to this population so that the number of infected is small. Therefore, we would recommend the use of DES to model the consequences of a new source of infection in an area. However, DES are rather complex and are unlikely to provide better results than System Dynamics Models for bigger cohorts. Agent-based simulations of cervical cancer are highly relevant when sexual behaviour differs between individuals and individuals can learn from experiences. This might be useful in some cases, but for most scenarios, System Dynamics Models would lead to the same results with less effort.

Furthermore, we conclude that we would need more emphasis put on the selection and formulation of mathematical models in the case of cervical cancer. The majority of models were published in journals of Medicine or Public Health, and very few in health economic or operations research journals. There is a risk that these non-economic journals put less emphasis on the appropriate model itself while focussing on medical or epidemiological correctness. Furthermore, model-builders and public health specialists should jointly reflect on the added value of different model options and the implications for public health practice. This multidisciplinary work would benefit policy and practice transfer.

In a nutshell: More research on epidemiological and economic processes is needed in the field of health economics and operations research to improve the robustness of models and respective results. Interdisciplinary groups composed of professional model-builders and public health experts are needed to develop these models and interpret the results. The global burden of cervical cancer warrants a joint search for the right type of model and its interpretation.

## Supplementary Information

Supplementary Material 1. Attachment: Key characteristics of the models [123–166].

## Data Availability

No datasets were generated or analysed during the current study.
