# Supplementary material for: Modelling epidemiological and economics processes – the case of cervical cancer
Source: Health Econ Rev. 2025 Feb 22;15:13. doi: 10.1186/s13561-024-00589-1 (PMC11846406; doi:10.1186/s13561-024-00589-1)
Supplement: Supplementary file 1 — Supplementary Material 1. Attachment: Key characteristics of the models [123–166]. [file 13561_2024_589_MOESM1_ESM.docx]

# Attachment: Key characteristics of the models

| **Author** | **Year** | **Source** | **Journal** | **Country** | **Type of Model** | **Rationale for model** | **Economic / epidemiological** | **Intervention** | **Outcomes^[[1]](#footnote-1)^** | **Study aim** | **Time horizon** |
| --- | --- | --- | --- | --- | --- | --- | --- | --- | --- | --- | --- |
| Kim et al. | 2007 | [123] | Med | Brazil | Hybrid: System dynamics and agent-based | No | Eco + epi | Vaccination of boys | Lifetime costs, life expectancy, cost/QALY | CE of vaccination | Life span |
| Elbasha et al. | 2007 | [106] | Med | USA | System dynamics | yes | Eco + epi | Vaccination | Incidence, prevalence, cost/QALY | CE of vaccination | 100 yrs |
| Diaz et al. | 2008 | [124] | Med | India | DES | No | Eco + epi | Vaccination, screening | Incidence, life expectancy gains, lifetime costs, ICER, cost/YLS | CE of vaccination and screening | Life span |
| Elbasha et al. | 2009 | [77] | Eco | USA | System dynamics | No | Eco | Vaccination | Cost utility, quality adjusted survival | CE of vaccination | - |
| Olsen and Jepsen | 2010 | [75] | Eco | Denmark | Agent-based | Yes | Eco + epi | Vaccination | Incidence, prevalence, cost/QALY | CE of vaccination | 30 |
| McLay et al. | 2010 | [73] | Eco | USA | Markov | No | Eco | Screening | Incidence, mortality, life years lost | CE of screening | 70 yrs |
| Shi et al. | 2011 | [125] | Med | China | Hybrid: System dynamics and Markov | No | Eco + epi | Screening | Mortality, cost-effectiveness, cost/YLS | CE of screening | Life span |
| Vanni et al. | 2012 | [126] | Med | Brazil | DES | Yes | Eco | Vaccination | Cost/QALY, years of life saved | CE of screening | - |
| van de Velde et al. | 2012 | [91] | Med | Canada | Agent-based | Yes | Epi | Vaccination | Incidence | Effectiveness of screening | 70 |
| Ribassin-Majed et al. | 2012 | [107] | Med | France | System dynamics | Yes | Epi | Vaccination | Prevalence, incidence | Effectiveness of vaccination | 70 |
| Blasio et al. | 2012 | [127] | Med | Norway | System dynamics | No | Epi | Vaccination, screening | Incidence, mortality | Effectiveness of vaccination | - |
| Sharma et al. | 2012 | [128] | Med | Thailand | DES | No | Eco | Vaccination, screening | Cost/YLS | CE of vaccination and screening | Life span |
| Demarteau et al. | 2013 | [129] | Med | Belgium | Markov | No | Eco | Vaccination, screening | ICER per QALY | CE of vaccination and screening | Life span |
| Brisson et al. | 2013 | [130] | Med | Canada | Agent-based | Yes | Eco | Vaccination | Cost/QALY | CE of vaccinations | 70 yrs |
| Kim et al. | 2013 | [72] | Med | EMENA region | Unclear | No | Eco + epi | Vaccination, screening | Incidence, mortality, DALYs, YLS, cost/DALY | CE of vaccination and screening | Life span |
| Vänskä et al. | 2013 | [131] | Med | Finland | System dynamics | No | Epi | Vaccination | Prevalence | Effectiveness of vaccination | - |
| Kim et al. | 2013 | [72] | Med | Sub-Saharan Africa | Non-simultaneous equation | No | Eco + epi | Vaccination, screening | Incidence, mortality, DALYs, YLS, cost/DALY | CE of vaccination | Life span |
| Drolet et al. | 2014 | [104] | Med | Canada | Agent-based | No | Eco | Vaccination | Cost/QALY | CE of vaccination | 70 |
| Gomez et al. | 2014 | [132] | Med | Chile | Markov | No | Eco | Vaccination, screening | Cost/QALY, Cost/YLS | CE of vaccination and screening | Life span |
| Kiatpongsan and Kim | 2014 | [133] | Med | Kenya, Uganda | Markov | No | Eco | Vaccination | ICER | CE of vaccination | Life span |
| Demarteau et al. | 2014 | [103] | Med | Nigeria | Markov | No | Epi | Vaccination, screening | Incidence | Optimal cervical cancer prevention strategy | - |
| Burger et al. | 2014 | [134] | Med | Norway | System dynamics | No | Eco | Vaccination of boys | Cost/QALY | CE of vaccination | Life span |
| Aguilar et al. | 2015 | [90] | Med | Honduras | Non-simultaneous equation | No | Eco + epi | Vaccination | Lifetime costs, incidence, mortality, cost/DALY | CE of vaccination | Life span |
| Matthijsse, van Rosmalen et al. | 2015 | [92] | Med | Netherlands | Agent-based | No | Epi | Role of acquired immunity | Immunity | Incidence | - |
| Li et al. | 2015 | [135] | Med | South Africa | Markov | No | Eco + epi | Vaccination, screening | Incidence, mortality, cost/QALY | CE of vaccination | Life span |
| Campos et al. | 2015 | [80] | Med | Uganda | DES | No | Epi | Screening test sensitivity | Sensitivity, screening coverage, loss to follow-up | CE of screening | Life span |
| Flessa et al. | 2016 | [74] | eco | Cambodia | System dynamics | No | Eco + epi | Treatment, screening, vaccination | YLL, cost/YLS | Total CE | 100 |
| Setiawan et al. | 2016 | [136] | Med | Indonesia | Markov | Yes | Eco + epi | Vaccination, screening | Cost, incidence, cost/QALY | CE of vaccination and screening | 88 |
| Brisson et al. | 2016 | [137] | Med | USA | System dynamics | No | Eco + epi | Vaccination | Incidence, Cost/QALY | CE of vaccination | 70 |
| Sharma et al. | 2016 | [138] | Med | Vietnam | hybrid: System dynamics and agent-based | No | Eco + epi | Vaccination of boys | Lifetime risk, averting genital warts, cost/QALY | CE of vaccination | - |
| Simms et al. | 2017 | [139] | Med | Australia | System dynamics | No | Eco | Screening | Cost/life-year saved | CE of screening | - |
| VõrNo et al. | 2017 | [140] | Med | Estonia | Markov | No | Eco | Vaccination, screening | Cost/QALY | CE of vaccination and screening | 88 |
| Kosen et al. | 2017 | [141] | Med | Indonesia | Systems dynamics | No | Eco + epi | Vaccination, screening | Incidence, cost, cost/QALY | CE of vaccination and screening | 100 |
| Campos et al. | 2017 | [142] | Med | Nicaragua | DES | No | Eco + epi | Screening | Lifetime risk and costs, life expectancy, cost/YLS | CE of screening | Life span |
| Ekwunife and Lhachimi | 2017 | [82] | Med | Nigeria | DES | No | Eco | Vaccination, screening | YLL, YLS, cost/DALY | CE of vaccination and screening | 90 |
| Burger et al. | 2017 | [143] | Med | Norway | DES | No | Eco + epi | Screening | Lifetime risk and cost, life expectancy, cost/QALY | CE of screening | Life span |
| Ortiz et al. | 2017 | [144] | Med | Puerto Rico | System dynamics | Yes | Eco + epi | Vaccination | Prevalence, Incidence, mortality, ICER | CE of vaccination | - |
| Campos et al. | 2017 | [81] | Med | Uganda | DES | No | Eco + epi | Screening | Lifetime risk and costlife expectancy, equity, ICER | Trade-offs between equity and efficiency | Life span |
| Campos et al. | 2017 | [76] | eco | Uganda | DES | No | Eco + epi | Screening | Lifetime risk and cost, life expectancy, cost/YLS | CE of screening | Life span |
| van Minh et al. | 2017 | [88] | Med | Vietnam | Non-simultaneous equation | No | Eco | Vaccination | Cost of treatment, cost/DALY | Costs and CE vaccination | - |
| van Kriekinge et al. | 2018 | [145] | Med | Malaysia | Markov | No | Eco + epi | Vaccination, screening | Incidence, death, QALYs | CE of vaccination and screening | Life span |
| Tay et al. | 2018 | [146] | Med | Singapore | Systems dynamics | No | Eco + epi | Vaccination | Incidence, mortality, ICER | CE of vaccination | 100 |
| Tay et al. | 2018 | [105] | Med | Singapore | Markov | No | Eco | Vaccination, screening | Cost/QALY | CE of vaccination and screening | Life span |
| Mezei et al. | 2018 | [70] | Med | Uganda | DES | No | Eco + epi | Screening | Risk, cost/YLS | CE of screening | Life span |
| Mahumud et al. | 2019 | [86] | Med | Australia | Non-simultaneous equation | No | Eco | Vaccination | Cost/DALY | CE of vaccination | Life span |
| Jiang et al. | 2019 | [85] | Med | China | Non-simultaneous equation | No | Eco | Vaccination | Cost/DALY prevented | CE of vaccination | Life span |
| Campos et al. | 2019 | [147] | Med | El Salvador | DES | No | Eco + epi | Screening | Lifetime risk, lifetime costs, life expectancy, cost/YLS | CE of screening | Life span |
| Lee et al. | 2019 | [148] | Med | Korea | Markov | No | Eco + epi | Vaccination, screening | QALY, LYS, cost savings | CE of vaccination | Life span or 95 yrs |
| Tang et al. | 2019 | [149] | Med | Taiwan | Markov | No | Eco | Vaccination, screening | Cost/QALY | CE of vaccination | Life span |
| Datta et al. 2019 | 2019 | [150] | Med | UK | System dynamics | Yes | Eco | Vaccination of boys and girls | Costs, QALY, threshold price per vaccine dose | CE of vaccination | 100 |
| Mahumud et al. | 2020 | [151] | Med | Bangladesh | Non-simultaneous equation | No | Eco | Vaccination | Cost/DALY | CE of vaccination | Life span |
| Campos et al. | 2020 | [152] | Med | El Salvador | DES | No | Eco + epi | Screening | Risk, life expectancy, lifetime cost, cost/YLS | CE of screening | Life span |
| Abbas et al. | 2020 | [84] | Med | Global | Non-simultaneous equation | No | Eco + epi | Vaccination | Incidence, mortality, DALY | CE of vaccination | 100 |
| Setiawan et al. | 2020 | [136] | Med | Indonesia | Markov | No | Eco + epi | Vaccination, screening | Incidence, mortality, QALYs | CE, budget impact | 75 |
| Brisson et al. | 2020 | [153] | Med | LMICs | Systems dynamics | No | Epi | Vaccination, screening | Elimination | Effectiveness of vaccination and screening | 100 |
| Canfell et al. | 2020 | [154] | Med | LMICs | Systems dynamics | Yes | Epi | Vaccination, screening, treatment | Mortality | Effectiveness of vaccination | 100 |
| Zhao et al. | 2020 | [155] | Med | Rural China | Markov | No | Eco + epi | screening | Incidence, mortality, QALYs, cost-effectiveness, ICER | CE of screening | 50 |
| Hall et al. | 2020 | [156] | Med | Tanzania | System dynamics | Yes | Epi | HIV preventive and therapeutic interventions | Incidence, mortality | Effectiveness of intervention | - |
| Laprise et al. | 2020 | [157] | Med | USA | Agent-based | No | Eco + epi | Vaccination of older age | Prevalence, cost/QALY | CE of vaccination | 100 |
| Vale et al. | 2021 | [158] | Med | Brazil | Markov | No | Eco | Screening | QALY, cost/QALY | CE of screening | - |
| Ma et al. | 2021 | [159] | Med | China | System dynamics | No | Eco + epi | Vaccination, screening | Prevalence, Cost/DALY | CE of vaccination and screening | 50 |
| PortNoy et al. | 2021 | [87] | Med | Ethiopia, India, Nigeria, Pakistan | Non-simultaneous equation | No | Epi | Vaccination | Incidence, mortality, DALY | Effectiveness of vaccination | Life span |
| Drolet et al. | 2021 | [160] | Med | India, Viet Nam, Uganda, Nigeria | System dynamics | No | Eco + epi | Vaccination | Incidence, number of doses needed, cost/DALY | CE of vaccination | 100 |
| Cody et al. | 2021 | [161] | Med | Japan | Systems dynamics | Yes | Eco + epi | Vaccination | Incidence, mortality, ICER, cost/QALY | CE of vaccination | 100 |
| Keane et al. | 2021 | [162] | Med | Malaysia | Systems dynamics | No | Eco + epi | Screening | mortality, CER | CE of screening | - |
| Phua et al. | 2021 | [163] | Med | Singapore | hybrid: System dynamics and agent-based | Yes | Eco | Vaccination | Cost/QALY | CE of vaccination | Life span |
| Termrungruanglert et al. | 2021 | [164] | Med | Thailand | Systems dynamics | Yes | Eco + epi | Vaccination, screening | Vaccination costs, treatment costs, QALYs | CE of vaccination and screening | 100 |
| Zhou et al. | 2022 | [89] | Med | China | Non-simultaneous equation | No | Eco | Vaccination | Cost of vaccination and treatment, DALYs, ICER | CE of vaccination | - |
| Man et al. | 2022 | [93] | Med | India | Agent-based | Yes | Epi | Vaccination | Prevalence, incidence, life time risk | Effectiveness of vaccination | 100 |
| Nguyen et al. | 2022 | [165] | Med | Papua New Guinea | Systems dynamics | No | Eco + epi | Screening, treatment | Mortality, cost/YLS | CE of screening and treatment | Life span |
| Bénard et al. | 2023 | [166] | Med | India, Viet Nam, Uganda, Nigeria | System dynamics | No | Epi | Vaccination | Incidence | Effectiveness of vaccination | 100 |

1. DALY: Disability Adjusted Life-Years lost. YLS: Years of Life Saved. YLL: Years of Life Lost; QALY: Quality Adjusted Life Years. ICER: Incremental Cost-Effectiveness Ratio. CE: Cost-effectiveness [↑](#footnote-ref-1)
